# Book Reviews

**Published:** 1917-05

**Authors:** 


### BOOK REVIEWS

Books mentioned may be inspected at and ordered through this office. So far as possible, books received in any month will be reviewed in the issue of the second month following. Pamphlets, quarterly and similar periodicals, reports, transactions, etc., will, as a rule, merely be mentioned.

**Medical Clinics of Chicago,** March 1917. Published bi-monthly by W. B. Saunders Co., Philadelphia, $8 per year.

**University of Buffalo,** 71st Annual Catalogue of Medical Dept.

One of the most interesting features of this catalogue is the light it throws on a phase of medical economics. The graduates of 1916 numbered 35. The undergraduate classes number respectively, 1917—60; 1918—29; 1919—41; 1920—66; special students 8. It will be seen that while considerable fluctuation occurs, no notable change has been due to the increased requirements, and that, on the whole, the attendance has not declined proportionately to that of the country as a whole.

**Antiprohibition Manual,** published by the National Wholesale Liquor Dealers' Assn. of America.

**Changes in the Pharmacopoeia and the National Formulary.** U. S. Public Health Service Bulletin No. 107, by Martin I. Wilbert.

**Buffalo State Hospital,** 46th Annual Report, Dr. Arthur W. Hurd, Supt., for 9 months ending June 30, 1916.

The number of patients increased from 2,142 to 2,199. 67 were discharged as cured, 59 as improved, 3 as not insane, 143 died. On the average, 75 patients were on parole. The yearly per capita cost was $167.54, farm and garden products having been raised to the amount of over $6000 and $9000 worth of articles having been made by patients. 48% of the patients were employed in some form of useful labor.

**General Education Board,** Report of Secretary, 1915-16.

Published by the Board, 61 Broadway, N. Y. Over 1½ millions have been dispensed during the year, ¼ million to medical schools.

**Anatomical Names,** Especially the Basle Nomina Anatomica ("BNA"). By Albert Chauncey Eycleshymer, B. S., Ph. D., M. D., Assisted by Daniel Martin Schoemaker, B. S., M. D. With Biographical Sketches, by Roy Lee Moodie, A. B., Ph. D. Octavo 764 pages, illustrated by numerous wood engravings and by two full-page plates in red and black. Extra muslin, $4.50 net. Wm. Wood & Co., N. Y.

The list of anatomic names, in Latin, even including regional designations, is, on the whole, surprisingly small, occupying 84 loosely printed pages. 180 pages are devoted to brief biographies of anatomists. The remainder of the work is in the form of an index which is also a more extended list of anatomic terms, including proper names and English terms.

---

**Handbook of Suggestive Therapeutics, Applied Hypnotism, Psychic Science.** Henry S. Munro, Omaha, C. V. Mosby Co., St. Louis. 4th edition, 486 pages, $4.50.

Some of the most valuable chapters in this work are those that might seem to be subsidiary. For example: The Guidance of the Sexual Instinct which, broadly viewed, gives rise to artistic athletic and inventive activities; Conservation of Energy; Abuse of Personality; Brutality of Frankness; Moral Stamina—A Therapeutic Power; Self Mastery as a Fine Art. The author is thoroughly practical in his use of suggestion, hypnotism, etc., and, so far as possible, conveys his personal mastery of these arts to his readers.

---

**Food and the Principles of Dietetics,** Robert Hutchison, M. D., F. R. C. P., London. Wm. Wood & Co., N. Y. 617 pages, with plates and diagrams. $4.00, 4th edition.

The earlier editions of this work have been well received and the present edition brings the subject to date and has been improved in many details. The illustrations are strikingly instructive. For example, the picture of a loaf of bread, while not immediately recognizable to an American on account of the difference in shape, shows the composition at a glance. The average reader will be surprised to learn that 2/3 of the volume is gas and that 40-50% by weight is water. The discussion of relative cost shows many divergencies from American prices and we are somewhat surprised to note that American cheese is rated at 6 pence per pound in England. The work includes both scientific and practical considerations, in considerable detail and is especially characterized by the inclusion of information which one would not criticize the author for omitting but which is of the greatest interest.

---

**The Newer Methods of Blood and Urine Chemistry,** R. B. H. Gradwohl, M. D., St. Louis, C. V. Mosby Co., St. Louis. 240 pages, 65 illustrations, 4 color plates, $2.50.

Two of the color plates are to illustrate the general use of the colorimeter. About a quarter of the work is devoted to blood, including the estimation of sugar, creatinine and creatin, uric acid, urea, non-proteid nitrogen, cholesterol, total nitrogen and chlorides. About the same amount is devoted to the urine, including the same analyses and also that of ammonia, sediments, bacteria, and the application of the phenolsulphonphthale in test. The remainder correlates the particular technical methods and interprets them clinically. A bibliographic list is appended to each chapter. This book well assembles what is known as to the newer methods of examination, really takes for granted what is sufficiently treated in older works, and displays great personal familiarity with the subject.

---

**Transactions of the 22d Annual Meeting of the American Laryngological, Rhinological and Otological Society,** held at White Sulprur Springs, May 5 and 6, 1916. Published by the Society, Dr. Wm. H. Haskin, Sec., 40 E. 41, N. Y. 442 pages, illustrated.

---

**The Perry's Victory Centenary, 1913.** Report of the State Commission, George D. Emerson, of Buffalo, Secretary. Printed by J. B. Lyon Co., Albany, 1916. 309 pages.

This is well illustrated, with portraits of the participants in the celebration and with pictures of historic places, ships, etc. The various addresses are included with a mass of historic papers of the utmost value. The work of the Commission which has naturally devolved largely on the Secretary, has been performed ably.

---

**Annual Report of the Directors of the American Telephone & Telegraph Co.,** for 1916.

In 1876, at the Centennial Exposition, the first telephone was shown to the public but attracted little attention. In 1884, the number of telephones in use in this country, slightly exceeded 100,000. In 1900, they numbered a little more than 600,000. In 1905, there were over 2,000,000. At about this time, competition reduced rates and increased efficiency and the growth has been rapid so that, at present, there are nearly 9,900,000 stations, or one to between 10 and 11 of the population. The physical investment is surprisingly small: about 71 millions, approximately divided as follows: telephones, 17 millions (less than $2 a set); real estate ½ million; office equipment, ¼ million; long distance plant 53½ millions. The stocks and bonds, etc., amount to about $600,000,000, approximately balanced by stocks of associated companies held, and by the physical equipment. The net earnings (including dividends and interest paid) amount to nearly $45,000,000, nearly 8%. The gross revenue is $270,000,000, about $30 per station, the expenses $190,000,000, about $20 per station, making the net revenue on this basis, $75,000,000. Last year, the exchange connections were 28,500,000 a day (about 3 per station) and the toll connections 889,000, or about 1 every 11 days for each station. All of this is highly interesting and the principal facts should be borne in mind.

---

**Progressive Medicine,** Edited by H .A. Hare and L. F. Appleman, Philadelphia, published by Lea & Febiger, quarterly, 6.00 per annum. Vol. 20, No. 1, March 1917, 319 pages, illustrated.

This number deals with surgery of the brain, head, neck and thorax; infectious diseases, especially meningitis and poliomyelitis; diseases of children and a review of laryngo-otology. The reviews are by Frazier, Miller, Ruhrah, Crandall and Coates, and maintain the high standards established.

---

**Annual Report of the Superintendent of the Poor of Buffalo,** (Louis J. Kenngott) for the year ending June 30, 1916.

Until May 1916, medical services were rendered by 10 district physicians paid nominal salaries for their duties and engaged in private practice. Since then, 4 full-time physicians have been employed. With this exception, (charged to the Health Dept.) the upkeep amounts to less than $15,000 for salaries and $2,000 for office expenses, etc. Non-medical public charity was dispensed to the amount of about $44,500 for groceries, $9,000 for coal, $1,800 for shoes, $1,782 for vacant lot cultivation. Burials cost $3,645, drugs $2,179, other outdoor medical relief about $250. $1,686 was paid the various hospitals for emergency ambulance calls. 160 persons were cared for in "Homes" at a total cost of $15,940; 4,573 in hospitals at a total cost of $160,000. Approximately, $184,000 was expended directly on account of medical and surgical needs, $55,000 for the relief of poverty. It will be noted that the administrative expense was about 1/15 of the total. A very favorable detail is that $8,397 was refunded by recipients of temporary aid, and $423 recovered from unworthy applicants. The number of heads of families assisted with out-door aid ranged from 580 in June to 881 in Feb., the rise and fall being quite gradual. On the other hand, the number of individuals sent to hospitals ranged only from 293 to 355 with little reference to season except that in July, the number was 762—unless this number includes those left over from the previous fiscal year. Of the 1514 families receiving out-door relief, the nationality, according to birth of heads of families which, obviously exaggerated the number of Americans in any possible racial sense, was as follows: American 33%, Polish 30%, Italian 15%, German 8%, Irish 3½% Canadian 3½%, English 2%, scattering 5%. The 4350 persons sent to hospitals were divided as follows: American 62%, Polish 11%, Italian 7%, German 5%, Irish 4%, Canadian 3%, Russian Jew 2.5%, scattering 5½%. The racial differences between dependency for ordinary poverty and for sickness and accident, are noteworthy.

---

**Plan of Military Instruction of the Clinical Club of Albany, N. Y.** Pamphlet, 52 pages, issued by a committee, Dr. Joseph A. Cox, Chairman.

This is endorsed by Surgeon General W. C. Gorgas who gives credit to the Club for the initiative, in a plan for local medical men to study the sanitary problems of their region, as they would arise in case of war. Beside containing various details as to normal military organizations and the general duties of medical officers, it consists of the working out of various assumed problems, as the mobilization of drugs, care of refugees, in camps and in available public and semi-public buildings, transportation of wounded, including available ambulances and substitutes, as motor cars, most eligible routes to insure rapidity and ease of transportation, with due regard to condition of pavements, etc. We trust that similar study of local problems will be made by the physicians of the various important towns in our territory and shall be glad to hear from those especially interested.

---

**Cataract,** Senile, Traumatic and Congenital. W. A. Fisher, M. D., Chicago; Chicago Eye, Ear, Nose and Throat Hospital, 119 pages, illustrated, $1.50.

The author succinctly emphasizes the following points as establishing the practical value of this treatise: 1: A new method of acquiring technique upon the eye ball with the aid of four week old kittens. 2: Discarding all kinds of eye specula and holding the lids away from the eye when operating the eye after injuries. 3: Dressing and treatment after cataract operations. 4: A modification of the Smith-Indian operation for cataract, making the removal of the lens safe and necessarily the operation of choice. 5: A method of treating injuries of the lens other than watchful waiting. 6: A systematic procedure for determining the treatment of congenital cataract.

---

**The Accuracy of Certified Causes of Death,** Reprint 363 from U. S. Public Health Service Reports. This is accompanied by a request from the Committee on Accuracy of Certified Causes of Death, Dr. H. Emerson, Chairman, under the auspices of the Statistical Bureau of the Metropolitan Life Ins. Co., Mr. Geo. H. Van Buren, Executive Secretary.

This gives the 189 Causes of Death of the International List, and a criticism by numbers, which is especially valuable in suggesting causes to be accepted without autopsy. The ideal classification of deaths must, for many diseases, recognize three factors: the nature of the pathologic process, the identity of the germ (animal or vegetable) producing it or some other essential etiologic factor, the location of the process. For example, pneumonia: What kind of a pneumonia is it from the standpoint of morbid anatomy? Is it a pneumococcus pneumonia or to some other bacterium or due to some essential cause, such as a chemic irritant or circulatory state not essentially involving a bacterium? Where is the pneumonic process located and what is its extent? A lobar pneumonia is not necessarily pneumacoccic while a lobular pneumonia or even a bronchitis may be. A pneumococcic process, strictly a pneumonia from the bacteriologic standpoint and even from the pathologic response may be located outside the lung. Another practical consideration is that, while autopsy is highly desirable, it is not practicable even in a considerable minority of cases. A diagnosis may be thoroughly established without autopsy and may be in doubt with autopsy, not only on account of incompetence of the examiner or practical obstacles to ideally thorough procedures but because the death is essentially due to conditions of physiology rather than of anatomy. It seems inevitable that diagnosis must often depend upon clinical judgment and in many instances, as for suicides, the exanthemata, etc., such a diagnosis is quite dependable, as is recognized by the Committee. Perhaps it would be well, instead of specifying too definitely what causes of death may or may not be entered without autopsy, to allow generally for clinical diagnosis and to leave a special space on certificates for it. Something may be said in favor of peculiar and indefinite statements as to cause of death. ''Old age'' is unquestionably too freely used and often for ages so slightly advanced as to be disconcerting to any one of middle life. Yet there are numerous instances in which it is strictly correct though not closely analytic and in which it is practically impossible to analyse more closely. Death may occur without opportunity for observation and even a necropsy will not determine exactly which of the ''atria mortis'' was the first to fail at the last. Ridicule such an expression if you please, but it is deliberately employed to describe an experience that we all have had. ''Old age'' is far more correct in many instances than bronchitis or obstipation or myocardiotis. It is often confusing to attempt to distinguish between primary and secondary causes of death. A condition may be secondary in point of time and yet the essential cause of death. Indeed, one may even go so far as to claim that many serious conditions are scarcely capable of killing directly, but only by their more or less regular complications.

